# Perforated appendicitis in Amyand's hernia with generalized peritonitis: a case report

**DOI:** 10.1093/jscr/rjag552

**Published:** 2026-07-21

**Authors:** Andrey A Sopuev, Niiazbek N Mamatov, Azamat T Atakoziev, Mairam E Ernisova, Bommidi Sraveen, Zhansaya B Telmanova, Beibarys E Amankeldin

**Affiliations:** Department of Hospital Surgery with the Course of Operative Surgery named after Academician M.M. Mamakeev, I.K. Akhunbaev Kyrgyz State Medical Academy, 92 Akhunbaev Street, Bishkek 720020, Kyrgyz Republic; Department of Hospital Surgery with the Course of Operative Surgery named after Academician M.M. Mamakeev, I.K. Akhunbaev Kyrgyz State Medical Academy, 92 Akhunbaev Street, Bishkek 720020, Kyrgyz Republic; Department of Hospital Surgery with the Course of Operative Surgery named after Academician M.M. Mamakeev, I.K. Akhunbaev Kyrgyz State Medical Academy, 92 Akhunbaev Street, Bishkek 720020, Kyrgyz Republic; Department of Hospital Surgery with the Course of Operative Surgery named after Academician M.M. Mamakeev, I.K. Akhunbaev Kyrgyz State Medical Academy, 92 Akhunbaev Street, Bishkek 720020, Kyrgyz Republic; Faculty of General Medicine, I.K. Akhunbaev Kyrgyz State Medical Academy, 92 Akhunbaev Street, Bishkek 720020, Kyrgyz Republic; Department of Epidemiology, Central Asian Institute for Medical Research, 13 Almaty Street, Esil District, Astana 010000, Kazakhstan; Scientific and Clinical Center of Surgery named after G.V. Tsoy, Astana Medical University, 49A Beybitshilik Street, Saryarka District, Astana 010000, Kazakhstan

**Keywords:** Amyand's hernia, appendicitis, peritonitis, appendectomy, herniorrhaphy

## Abstract

Amyand’s hernia is an uncommon inguinal hernia containing the vermiform appendix. Preoperative diagnosis remains difficult, and many cases are recognized only during surgery. We report a 64-year-old man with a 33-year history of a reducible right inguinal hernia who presented with 5 days of irreducibility, groin pain, fever, and diffuse abdominal pain. Examination showed generalized peritonitis. Ultrasonography demonstrated a fluid-filled right inguinal hernia sac containing a tubular structure, free intraperitoneal fluid, and dilated bowel loops. Emergency inguinal exploration released purulent fluid and revealed the cecum with a gangrenous perforated appendix inside the hernia sac. Because purulent material tracked freely through the internal ring, the procedure was immediately converted to lower midline laparotomy. Appendectomy, abdominal lavage, drainage, and tissue-based hernia repair without mesh were performed. The patient recovered well and was discharged on postoperative day 16.

## Introduction

Amyand’s hernia is a rare inguinal hernia in which the appendix lies within the hernia sac [1]. Most cases are diagnosed intraoperatively because the presentation usually resembles an incarcerated or strangulated groin hernia rather than primary appendiceal disease [1–4]. Management is individualized and depends mainly on the condition of the appendix and the degree of contamination [2, 7, 8]. We report a perforated Amyand’s hernia complicated by generalized fibrinopurulent peritonitis, in which intraoperative recognition of purulent communication through the internal ring dictated immediate escalation from groin exploration to laparotomy for definitive source control.

## Case report

A 64-year-old man was admitted emergently with a painful irreducible right groin mass, fever, and progressive diffuse abdominal pain. He had a 33-year history of a reducible right inguinal hernia that became irreducible 5 days before admission. His only reported comorbidity was grade 2 arterial hypertension.

On examination, body temperature was 38.9°C, heart rate 102 beats/min, blood pressure 150/90 mmHg, and respiratory rate 21/min. A tense tender right inguinal mass measuring ~6.0 × 5.0 cm was present with erythema of the overlying skin. The abdomen was distended and diffusely tender with peritoneal signs. Laboratory testing showed leukocytosis and markedly elevated procalcitonin (Table 1).

**Table 1:** Laboratory findings on admission and at discharge.

Parameter	On admission	At discharge	Reference range
WBC (×10^9^/L)	17.3	6.3	4.0–9.0
ESR (mm/h)	28	—	<15
Procalcitonin (ng/mL)	34.23	0.12	<0.5
CRP (mg/L)	8.5	—	<5.0
Glucose (mmol/L)	10.8	—	3.9–6.1

Ultrasonography demonstrated a fluid-containing right inguinal hernia sac with a tubular structure and surrounding fluid. Abdominal ultrasonography showed free intraperitoneal fluid and dilated small-bowel loops. Because clinical examination already demonstrated generalized peritonitis, computed tomography was deferred to avoid delaying definitive source control.

**Figure 1:**
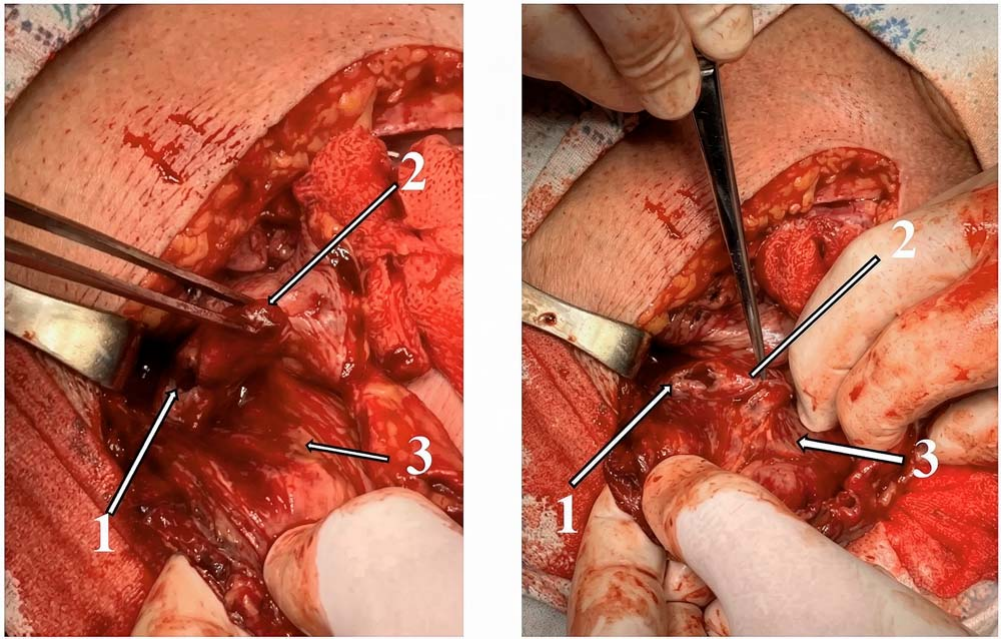
Intraoperative findings during emergency right inguinal exploration: (1) gangrenous appendix with perforation; (2) distal appendix within the hernia sac; (3) thickened hernia sac containing the cecum and appendix.

Emergency surgery began through a right inguinal oblique incision. The hernia sac was thickened and covered with fibrinopurulent exudate. After opening the sac, ~300 mL of foul-smelling purulent fluid was released. The cecum and a gangrenous perforated appendix were identified within the hernia sac (Fig. 1). Digital assessment of the internal ring demonstrated free communication of pus with the peritoneal cavity. The operation was therefore converted immediately to lower midline laparotomy.

Approximately 1000 mL of purulent fluid was evacuated from the abdominal cavity. Generalized fibrinopurulent peritonitis with multiple interloop abscesses and pelvic collections was found. Appendectomy, extensive warm saline lavage, and drainage of the pelvis and right paracolic gutter were performed. Because the field was grossly contaminated, the posterior wall of the inguinal canal was repaired using a tissue-based Bassini technique without mesh.

Postoperatively, the patient received ceftriaxone, metronidazole, infusion therapy, and close monitoring. Bowel function recovered on postoperative day 4. Drains were removed between postoperative days 5 and 9, and delayed secondary sutures were placed in the groin wound on postoperative day 13. The patient was discharged in stable condition on postoperative day 16. Histopathology confirmed gangrenous appendicitis with perforation. At 1-month follow-up, he had no fever, no recurrent groin swelling, and had returned to baseline activity. Written informed consent for publication was obtained.

## Discussion

This case illustrates two practical points. First, Amyand’s hernia may masquerade as an incarcerated inguinal hernia even when the true pathology is perforated appendicitis with diffuse abdominal sepsis. Second, the decisive operative finding is not only the appendix within the sac, but also whether septic contamination extends beyond the groin. In the present patient, free purulent tracking through the internal ring meant that groin access alone could not achieve source control.

Cross-sectional imaging can sometimes identify Amyand’s hernia preoperatively and may help in stable patients [5]. Here, however, generalized peritonitis was already evident clinically and urgent surgery took priority. This approach is consistent with emergency hernia practice, in which delay for additional imaging is difficult to justify when the need for source control is already clear [6].

The choice of hernia repair in contaminated Amyand’s hernia remains debated. Recent reviews and guideline updates emphasize individualized decision-making, especially when perforation, pus, or dirty-field conditions are present [2, 7]. Evidence comparing mesh with non-mesh repair in emergency groin hernia surgery remains uncertain, particularly in contaminated settings [8]. Given the perforated appendix, diffuse pus, and multiple abscesses, tissue repair without mesh was the safest option in this case.

The markedly elevated procalcitonin level supported the overall impression of severe bacterial sepsis. Although procalcitonin is not sufficiently accurate to diagnose appendicitis by itself, it has greater value in complicated appendicitis than in uncomplicated disease [9]. The main limitation of this report is that it describes a single patient with short follow-up. Even so, the case underlines an important rule: when groin exploration reveals perforated appendicitis with purulent communication through the internal ring, prompt conversion to laparotomy may be required for definitive abdominal source control.

## Data Availability

All relevant clinical data are contained within the article.
